# Prevalence of endoepicardial asynchrony and breakthrough patterns in a bilayer computational model of heterogeneous endoepicardial dissociation in the left atrium

**DOI:** 10.1371/journal.pone.0314342

**Published:** 2024-11-22

**Authors:** Elham Zakeri Zafarghandi, Vincent Jacquemet

**Affiliations:** 1 Department of Pharmacology and Physiology, Faculty of Medicine, Université de Montréal, Montreal, Quebec, Canada; 2 Research Center, Hôpital du Sacré-Coeur de Montréal, Montreal, Quebec, Canada; Institute for Basic Science, REPUBLIC OF KOREA

## Abstract

**Background:**

Transmural propagation and endoepicardial delays in activation observed in patients with atrial fibrillation are hypothesized to be associated with structural remodeling and endoepicardial dissociation. We aim to explore in a computational model how the distribution of delays and the rate of endo- and epicardial breakthrough activation patterns are affected by fibrosis and heterogeneous layer dissociation.

**Methods:**

A bilayer interconnected cable model of the left atrium was used to simulate a total of 4,800 episodes of atrial fibrillation on 960 different arrhythmogenic substrates with up to 30% epicardium-only diffuse fibrosis. Endoepicardial connections were heterogeneously distributed following random spatial patterns (characteristic length scale from 1.6 to 11.4 mm). Intermediate nodes were introduced in the transmural connections to enable the simulation of weaker coupling. This heterogeneous interlayer dissociation divided the atrial bilayer into connected and disconnected regions (from 27 to 48,000 connected regions). Activation time series were extracted in both layers to compute endoepicardial delays and detect breakthrough patterns.

**Results:**

Because of epicardial fibrosis, fibrillatory waves were driven by the endocardium, which generated endoepicardial delays. The delays in the connected regions (up to 10 ms, but generally < 5 ms) were prolonged by higher fibrosis density and weaker coupling. Disconnected regions allowed longer delays (> 15 ms) and promoted the occurrence of breakthroughs. These breakthroughs had short lifespan (< 10–20 ms) and were more prevalent with higher fibrosis density and heterogeneous dissociation (larger disconnected regions). Severe remodeling (< 500 connected regions) was needed to produce clinically reported rates (> 0.1 breakthrough/cycle/cm^2^).

**Conclusion:**

Heterogeneous endoepicardial dissociation aggravates activation delays and increases the prevalence of epicardial breakthroughs.

## 1. Introduction

Atrial fibrillation (AF) is an increasingly prevalent arrhythmia that slowly progresses from its acute form to its chronic form through the remodeling of the cardiac tissue [1–3]. This remodeling process involves persistent changes in cellular electrophysiology (down- and up-regulation of ion channels), intracellular calcium handling, gap junction function, regulation by the autonomic nervous system, atrial chamber size and strain, and in conduction disturbances resulting from the development of fibrosis [2]. In particular, structural remodeling may interrupt the continuity of fiber bundles and laterally separate groups of fibers [2], create regions of slow conduction and zig-zag propagation patterns [4], provide a substrate for micro-reentries [5, 6], strengthen anisotropic conduction in aging tissue [7], induce loss of continuity in the thin epicardial layer due to endomysial fibrosis [8], and promote electrical dissociation between the endo- and the epicardial layers [9]. Possible clinical manifestations of endoepicardial dissociation are the emergence of long endoepicardial delays in electrical activation and the occurrence of breakthrough activation patterns [10].

Endoepicardial delays have been documented as early as in 1971 in canine atria [11]. Subsequent studies built upon the technological improvements of simultaneous endo- and epicardial optical [6, 12] and electrical mapping systems [13–15]. Delays up to 2 ms [11] or ±6 ms [14] have been reported in canine right atria in sinus rhythm, and up to ±12 ms in AF [14]. More recent investigations demonstrated the occurrence of larger endoepicardial asynchrony in persistent AF patients, with delays sometimes exceeding 15 or 20 ms [16–18]. Concomitantly, breakthrough activation patterns have been observed [12, 19, 20], even in sinus rhythm [21, 22]. These evidences support the hypothesis of a three-dimensional substrate of AF [6, 23]. The trabecular structure of the right atrium certainly facilitates transmural gradients [24], but dyssynchrony has also been observed in the left atrial wall [18]. It remains unclear which mechanisms explain how a relatively thin atrial wall can sustain large transmural asynchrony.

Computational models have been developed to investigate these mechanisms by introducing electrophysiological differences between the epicardial and the endocardial layer. One group of studies created a limited number of discrete and distant endoepicardial connections or partly connected endocardial bundles [12, 25–29]. The sparseness of the connections between two otherwise uncoupled layers enabled three-dimensional reentries within the wall, which in each layer was observed as breakthrough activation. This increased arrhythmogenicity as compared to a single layer model [26]. Another group of studies included epicardial fibrosis, which created a transmural gradient in conduction properties and consequently an activation delay [21, 27, 30].

In this paper, we present a computational model of the left atrium that combines epicardial fibrosis with weak, sparse and heterogeneous endoepicardial coupling, thus covering the range between the two groups of previous studies. This model was constructed from a bilayer interconnected cable structure which facilitated the separate, precise control over intralayer and interlayer coupling. We investigated the conditions that promote endoepicardial delays and breakthroughs during sinus rhythm and AF. Under the hypothesis of heterogeneous endoepicardial dissociation within a thin wall of uniform thickness, we estimated the parameters that would be needed to reproduce the experimentally measured prevalence of transmural asynchrony and breakthroughs.

## 2. Materials and methods

### 2.1. A bilayer model of the left atrium

**Fig 1:**
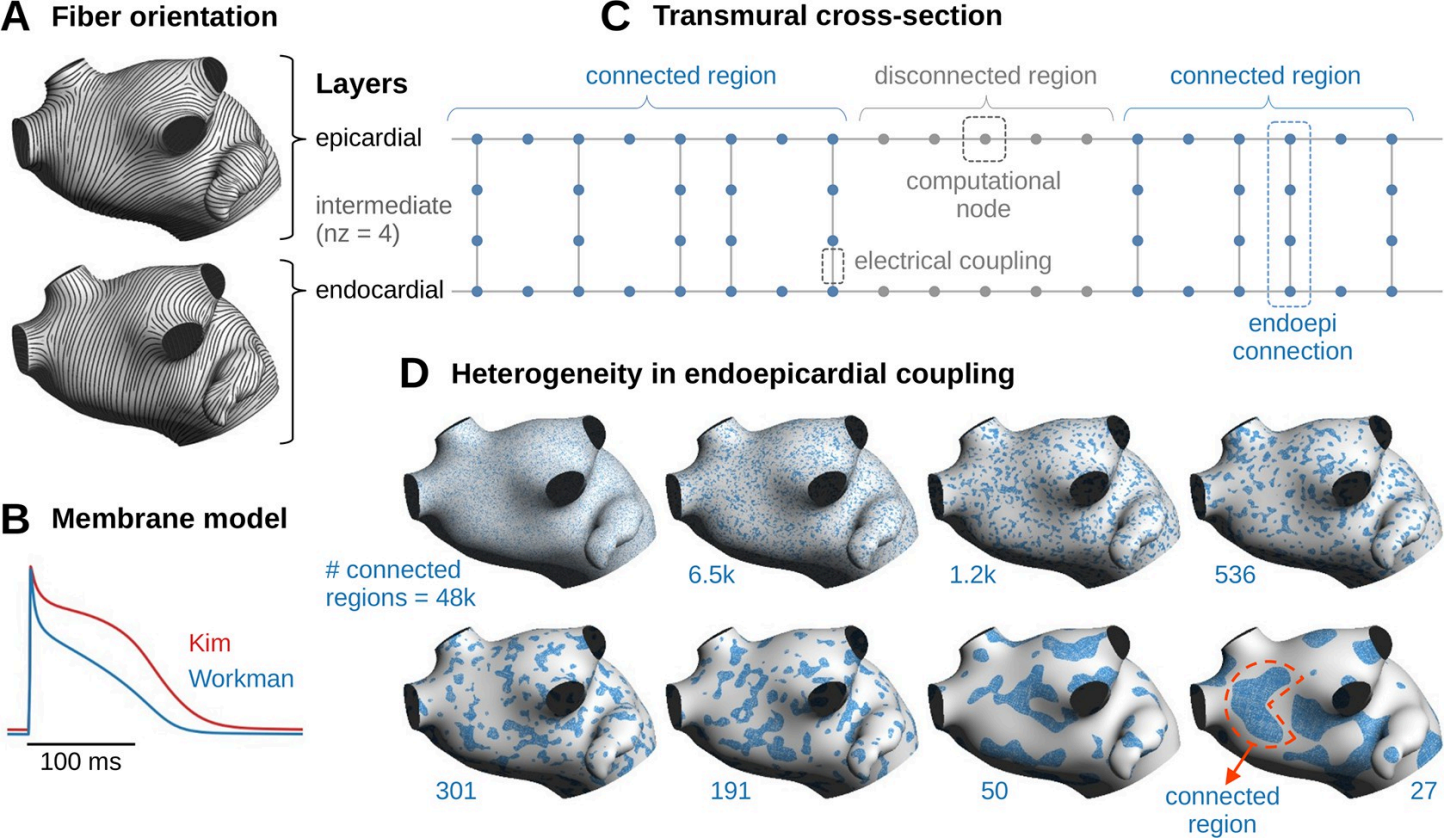
Construction of the atrial model. (A) Fiber orientation in the epicardium (top) and in the endocardium (bottom) from which longitudinal cables were generated. (B) Action potentials simulated using the two membrane models during paced rhythm at 200 bpm. (C) Illustration of a transmural cross-section of the cable model showing the epi- and endocardial layers and the transmural cables with n_z_ = 4. The color (blue/gray) of the nodes corresponds to those of the patterns below. (D) Examples of random realizations of endoepicardial coupling patterns with increasing length scale. The number of connected regions is indicated below each pattern. The number of transmural cables is, however, the same in all patterns.

The interplay between the electrical activation in the endo- and in the epicardium was investigated in a bilayer interconnected cable model of the human left atrium described in our previous works [31–33]. The geometrical mesh was a cable network composed of 8635 cables organized in two layers and aligned with fiber orientation (Fig 1A), and 9610 orthogonal cables creating transverse connections between longitudinal cables, for a total of 1.9 million nodes with a spatial resolution of 100 μm. Mutual nearest neighbors between the two layers were connected through transmural cables (see Section 2.3). The bilayer geometry should be viewed in the light of the formalism of Coudière et al. [34] expressing the small-thickness limit of a two-layer slab of myocardium. The atrial wall is assumed to be composed of two layers with different properties, each one ~1–2 mm in thickness and with uniform properties along the transmural axis. In this formalism, information about wall thickness has been shown to be encoded in the transmural coupling of the equivalent bilayer model [34].

Electrical propagation was simulated using the monodomain equation with two remodeled variants of the Courtemanche *et al*. [35] atrial cell model (Fig 1B). The first variant, referred to as the “Workman” model, consisted in 63% I_CaL_ inhibition, 65% I_to_ inhibition and 73% increase in I_K1_, based on experimental measurements by Workman *et al*. [36]. The second variant, referred to as the “Kim” model, consisted in 30% I_CaL_ inhibition, 80% I_to_ inhibition, 90% I_Kur_ inhibition, and 50% increase in I_Kr_, and was designed to reproduce the relatively steep clinical restitution curves obtained by Kim *et al*. [37] in human atria. The two membrane models generated different fibrillatory dynamics: stable rotors for the Workman model and meandering waves for the Kim model [33].

Numerical methods, presented in more details in Saliani *et al*. [31], exploited the cable structure of the model to improve efficiency [38, 39]. The time step was 50 μs for diffusion and 12.5 to 100 μs for membrane kinetics. Propagation in our cable model has been previously validated against a thin-walled cubic mesh [40].

### 2.2. Endo- and epicardial conduction properties

The longitudinal and transverse conductivity in each layer was set to 4 and 1 mS/cm respectively. Structural differences between the layers were introduced to create endoepicardial delays in propagation. Diffuse fibrosis was implemented as random decoupling of both longitudinal and transverse connections. Epicardial fibrosis density was set to 0%, 10%, 20% or 30%. There was no fibrosis in the endocardial layer. This configuration was inspired by the experimental observations of Verheule *et al*. [8] and the simulations of Gharaviri *et al*. [21].

### 2.3. Endoepicardial coupling

Endoepicardial dissociation is expected to increase propagation delays and promote the occurrence of breakthroughs [9, 17]. Weak, sparse and heterogeneous endoepicardial coupling was introduced to model layer dissociation. A total of 181,608 transmural cables connecting corresponding endo- and epicardial nodes were created. This is a reduction of 75% as compared to our maximally coupled model [31].

In the thin wall limit of a bilayer model, interlayer coupling reflects both wall thickness and transmural conductivity σ_z_ [41]. If that coupling is too low, there is a risk of conduction block (numerical artifact) caused by the tissue passive space constant becoming smaller than the space step Δz. The proposed solution is to add intermediate active nodes in the transmural cables between the endo- and the epicardium (Fig 1C). Instead of two, transmural cables have n_z_ ≥ 2 nodes. Physiologically, transmural cables might be interpreted as isolated zigzag pathways that percolate through a fibrotic midmyocardium [42]. The total passive endoepicardial coupling produced by one cable is proportional to σ_z_/((n_z_-1)Δz), so increasing n_z_ is equivalent to reducing σ_z_ in that aspect. To cover a wide range of low coupling values, the conductance σ_z_ was set to 0.2, 0.3 or 0.4 mS/cm and n_z_ was set to 2, 4, 6, 8 or 10, while Δz was kept constant at 100 μm like within the layers for consistency. This means that increasing n_z_ did not improve numerical convergence but rather impaired transmural conduction by extending the activation delay along the transmural pathway of the endoepicardial connections. The resulting number of computational nodes increased to up to 3.35 million due to the intermediate nodes.

Endoepicardial coupling was made heterogeneous by spatially distributing those 181,608 transmural cables into clusters that we will call connected regions (blue regions in Fig 1D). For that purpose, random spatial patterns were generated using a Gaussian-filtered random field quantized by thresholding [43]. Varying the length scale of the spatial filter changed the number and the average size of the connected regions (examples of realizations shown in Fig 1D). The number of connected regions, identified as the number of connected components of the patterns, was chosen as a control parameter to facilitate the comparison with the study by Gharaviri et al. [26], and ranged from 27 to 48k (8 values indicated on Fig 1D). In the gray regions of Fig 1D, the endo- and epicardial layers are dissociated as illustrated in Fig 1C. For regression analysis, a more convenient quantifier of heterogeneity is the length scale defined as R_region_ = (A_atrium_/πN_conn_)^1/2^, where A_atrium_ is the area of the left atrium (101 cm^2^) and N_conn_ is the number of connected regions. The values of R_region_ ranged from 0.26 to 10.9 mm.

### 2.4. Simulation protocol

Sinus rhythm was simulated by injecting intracellular current in the Bachmann’s bundle region. Atrial fibrillation was initiated by generating five fibrillatory initial conditions (each with one phase singularities) using an eikonal-diffusion approach [44, 45]. The simulations were run for one second to stabilize the fibrillatory dynamics, and then for five additional seconds on which the analyses were performed. Initiation of fibrillation was considered successful if reentrant activity was still present after one second. The criterion for self-termination of fibrillation was all membrane potentials being lower than -60 mV.

The protocol followed a full factorial design with 2 membrane kinetics × 4 levels of epicardial fibrosis density × 5 values of n_z_ × 3 values of σ_z_ × 8 spatial patterns × 5 initial conditions, which gave a total of 4,800 simulations in atrial fibrillation and 4,800 in sinus rhythm (only the resting initial condition is used in sinus rhythm, but 5 statistical realizations of each substrate were generated to balance the sample sizes).

### 2.5. Endoepicardial delays

Activation times were identified as time instants when the membrane potential crossed -40 mV with a positive slope. They were calculated at every node of the mesh. At 12,669 points uniformly spread over the atrial surface, matching time series of activation in the endo- and epicardium were extracted for further analysis. It was determined whether these points were in the connected regions (within 1 mm from an endoepicardial connection) or in the disconnected regions for statistical comparison. The delays between corresponding activation in the endo- and epicardium were computed using the mutual nearest neighbors between the two time series [33]. By convention, a positive delay meant that the endocardium was activated earlier.

To calculate aggregate statistics, delays from all sites or from the sites in the connected/disconnected regions were pooled. Kernel-density estimation of the delay distribution was used to determine the mode of the delay (most probable value).

### 2.6. Breakthrough detection

In each layer, wavelets were defined as topologically connected regions with membrane potential higher than -40 mV. The threshold was set sufficiently high to prevent detection of non-propagating passive response (false positives). These wavelets were tracked over time with a temporal resolution of 1 ms. Emerging wavelets with no intersection with any wavelet from the preceding time step were considered breakthroughs. Their lifespan was determined, as well as the maximum size (in number of nodes) they reached [28]. To facilitate the comparison with simultaneous endoepicardial mapping systems with inter-electrode distance of 2 mm [17], only breakthroughs that propagated enough to spread over 400 computational nodes (equivalent to 20 × 20 nodes with 0.1 mm space step) were considered potentially experimentally observable by non-punctual extracellular electrodes and were kept for further analyses.

The incidence of breakthroughs in #/cycle/cm^2^ was defined as the number of detected breakthroughs divided by the number of cycles during the interval of analysis and by the atrial surface area (101 cm^2^). The number of cycles was one per beat during sinus rhythm and was calculated as the duration of simulated fibrillation divided by the median cycle length during that episode of fibrillation.

The prevalence of breakthroughs in #/cm^2^ was defined as the average number of breakthrough waves present at a given time instant divided by the atrial surface area.

### 2.7. Statistical analysis

Data analyses were performed in python using the packages ‘scipy.stats’ for t-tests and ANOVA, ‘statsmodels’ for linear and logistic models, and ‘lifelines’ for Kaplan-Meier survival curves and Cox proportional hazards regression. Confidence intervals were computed at 95% confidence level.

## 3. Results

### 3.1. Simulation of sinus rhythm propagation

The total activation time of the left atrial endocardium during sinus rhythm was measured for each simulation. Wave front propagation was driven by the faster endocardial layer and slowed down by the current flowing to the epicardial layer. Propagation was slower with higher fibrosis density and stronger layer dissociation. The total activation time was 170.1 ± 6.4 ms with the Workman model and 162.6 ± 5.9 ms with the Kim model. Multiple linear regression with ordinary least squares (n = 4800, R^2^ = 0.76) gave an intercept of 151.5±0.2 ms (corresponding to the Kim model with no fibrosis, n_z_ = 2 and σ_z_ = 0.2 mS/cm), plus 7.5±0.1 ms when the Workman model was used, 3.63±0.05 ms for each 10% increment of epicardial fibrosis, 0.78±0.02 ms for each additional intermediate node (n_z_), and 2.45±0.06 ms for each increment of 0.1 mS/cm of transmural conductivity. The p-value was < 0.005 for all independent variables. It appears that the faster endocardial wavefront was slowed down by the electrotonic load from the epicardial layer in which conduction was impaired.

**Fig 2:**
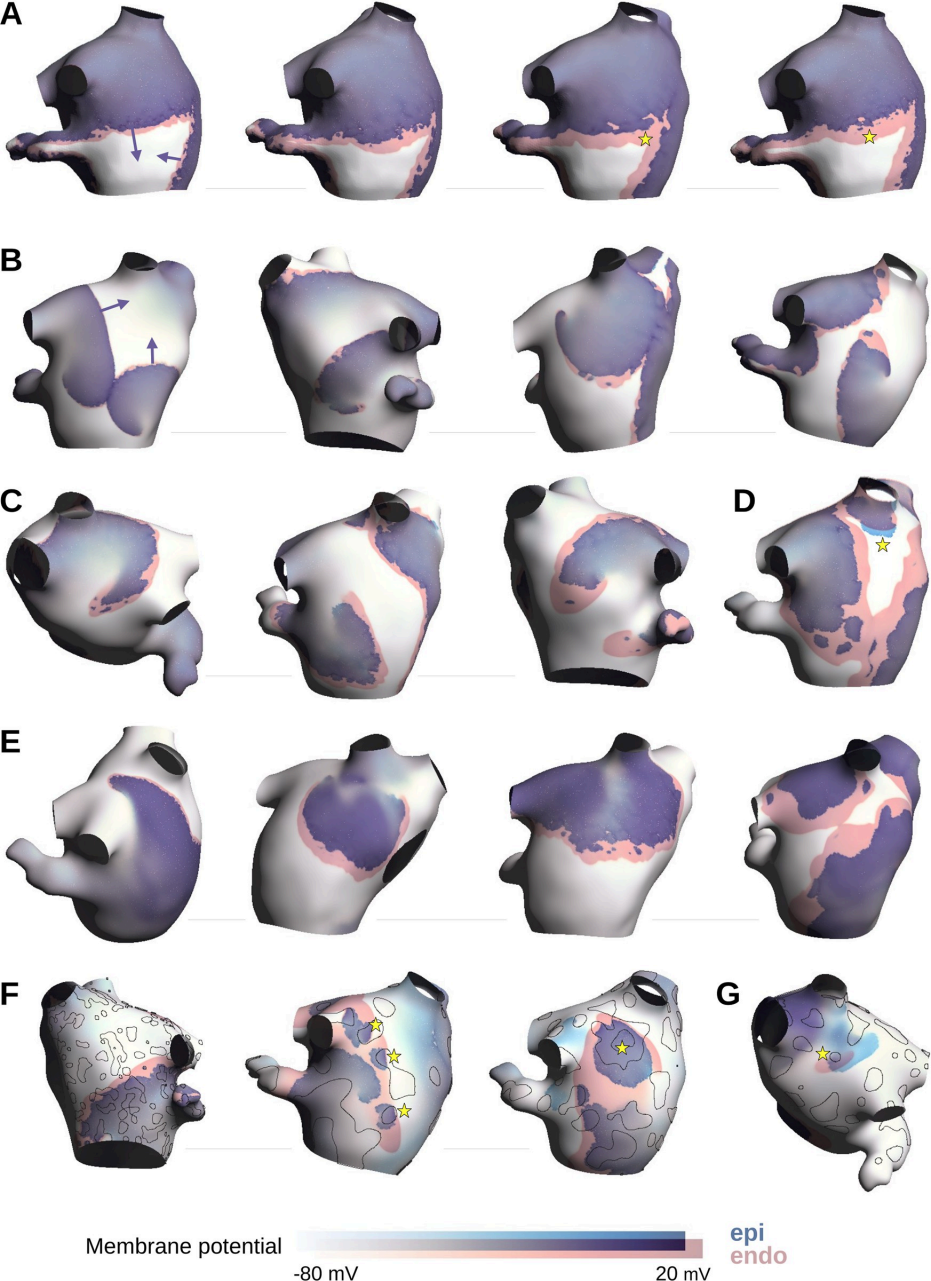
Examples of membrane potential maps during sinus rhythm (A) and atrial fibrillation (B-G) in arrhythmogenic substrates with increasing levels of endoepicardial dissociation. Membrane potential maps in the epicardium (blue color map) and in the endocardium (pink color map) were blended with transparency on each atrial surface to highlight the delay between the activation of the two layers. Arrows indicate direction of propagation. Yellow stars are referred to in the text. The Workman model was used in all panels except panels E and G. In panels F and G, the contours of the connected regions as shown in Fig 1 are outlined in black. Parameter sets encoded as a quadruple (n_z_, #connected regions, σ_z_, fibrosis density) were as follows: Panel A, from left to right: (4, 1.2k, 0.4, 30%), (6, 536, 0.2, 30%), (10, 301, 0.2, 20%), (10, 1.2k, 0.2, 30%); Panel B: (2, 6.5k, 0.4, 20%), (4, 1.2k, 0.4, 30%), (2, 301, 0.4, 20%), (2, 191, 0.4, 20%); Panel C: (4, 536, 0.3, 30%), (6, 536, 0.2, 30%), (10, 191, 0.2, 30%); Panel D: (10, 50, 0.2, 30%); Panel E: (4, 6.5k, 0.3, 30%), (10, 1.2k, 0.3, 20%), (6, 536, 0.3, 30%), (6, 27, 0.2, 20%); Panel F: (10, 191, 0.4, 30%), (4, 27, 0.2, 30%), (6, 50, 0.2, 30%); Panel G: (10, 50, 0.2, 20%).

The shape of the wavefronts during sinus rhythm is illustrated in Fig 2A. The epicardial waves (blue) were delayed with respect to the endocardial waves (pink). With increasing number of intermediate nodes and decreasing number of endoepicardial connections (from left to right in panel A), the delay became longer, and breakthroughs began to occur (yellow stars), helped by the irregularity of epicardial wavefronts caused by fibrosis.

To quantify how fibrosis slowed down propagation when layers were dissociated, propagation in fully uncoupled epi- and endocardium layers was simulated. The ratio of average conduction velocity (CV_ratio_) was estimated as the ratio of activation times in the epi- and in the endocardium. For 0%, 10%, 20% and 30% epicardial fibrosis, CV_ratio_ was respectively 1.03, 1.24, 1.58 and 2.13 with the Workman model and 1.03, 1.24, 1.56 and 2.06 with the Kim model. The ratio at 0% is explained by endoepicardial differences in fiber orientation. The parameter CV_ratio_ will be used later for regression analysis.

### 3.2. Simulation of atrial fibrillation episodes

Out of the 3,600 attempts to initiate AF in a substrate with fibrosis, 1,944 (81%) were successful with the Workman model and 677 (28%) with the Kim model. In the absence of fibrosis (another 1,200 attempts), these percentages dropped to 62% and 3% respectively. Logistic regression showed that increased fibrosis and additional intermediate nodes (n_z_) promoted AF inducibility (p<0.005). The effect of transmural conductivity was modest because a limited range of low conductivity values were considered. Note that the factors that prolonged total activation times and therefore reduced average conduction velocity also facilitated AF inducibility.

**Fig 3:**
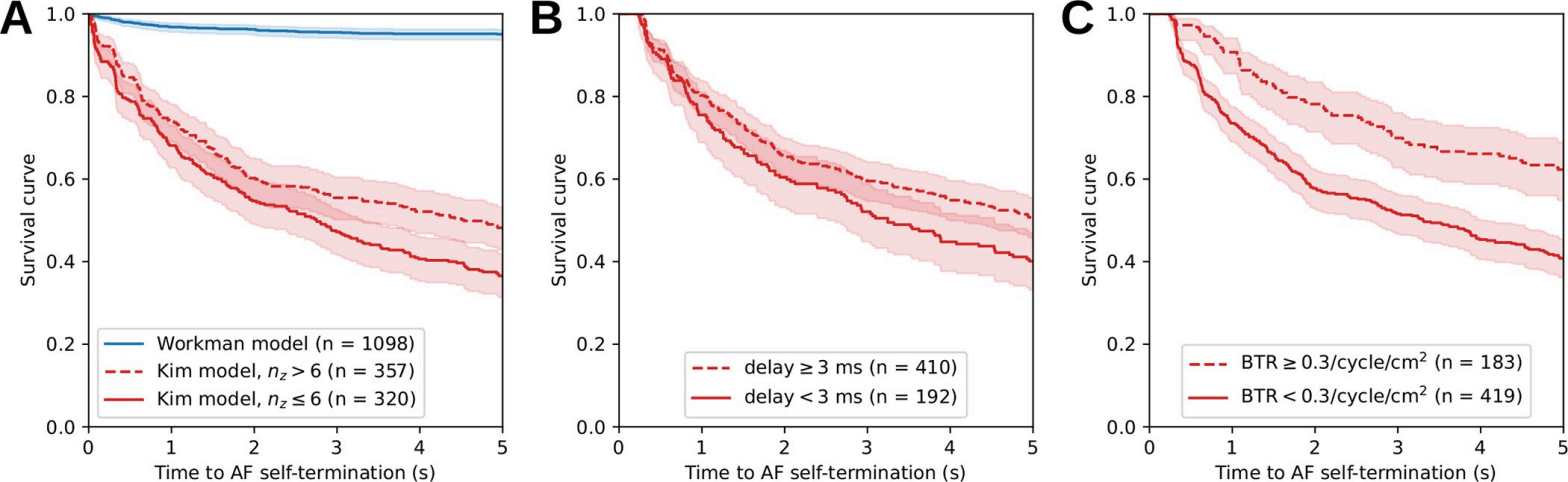
Occurrence of self-termination in simulated episodes of atrial fibrillation (AF), represented by Kaplan-Meier estimates of the survival curve with its confidence intervals displayed as light shaded regions. (A) The simulations with the Kim model were divided into two subgroups depending on the number of intermediate nodes n_z_ being smaller or larger than 6. The hazard ratio between the two subgroups was 0.74 (confidence interval: 0.61 to 0.90) as obtained by Cox regression (p<0.005). The simulations using the Workman model are displayed as a single group. (B) Survival curves for the Kim model divided into two subgroups where the mode of the endoepicardial delay distribution is smaller or larger than 3 ms. (C) Survival curves for the Kim model with two subgroups formed using a threshold on the breakthrough rate (BTR). Note that in panels B and C, a minimum AF duration is needed to estimate the quantities, so the sample size is smaller than in panel A.

Simulated AF was observed as a stable rotor (Workman model) or as meandering waves (Kim model), thus providing two very different types of fibrillatory dynamics [33]. While rotors most often led to sustained AF, meandering waves tended to self-terminate more frequently, as illustrated by the survival curve for successfully initiated AF episodes (Fig 3A). We did not observe conversion to atrial flutter in this left atrial model within our range of parameter values. Cox proportional hazard regression was performed on the Kim model with fibrosis. Increased fibrosis and additional intermediate nodes (n_z_) both prolonged AF duration (p<0.005), while the effect of transmural conductivity was not convincingly significant (p = 0.05). The hazard ratio was multiplied by a factor 0.82 (confidence interval: 0.72 to 0.92) for each additional 10% of fibrosis and by a factor 0.92 (confidence interval: 0.89 to 0.96) for each additional intermediate node. With the Workman model, only fibrosis had a significant effect (p = 0.02) despite the large number of observations (n = 1,944), suggesting that once a rotor was robustly established, it was sustained.

Cox regression was then applied to test the effect of endoepicardial delay (see section 3.3) and breakthrough rate (see section 3.4) with the Kim model. A mode of the delay ≥ 3 ms was associated with longer AF duration (hazard ratio of 0.76; confidence interval 0.60 to 0.95; p = 0.02; Fig 3B). However, the delay treated as numerical variable was not found to be a significant predictor of AF duration (p = 0.34), which deemphasizes the effect of the delay. A larger breakthrough rate in #/cycle/cm^2^ was associated with longer AF duration (p<0.005). The hazard ratio was multiplied by a factor of 0.87 (confidence interval: 0.82 to 0.92) for each increase by 0.1/cycle/cm^2^ of the breakthrough rate. Survival curves with a cutoff at 0.3/cycle/cm^2^ are presented in Fig 3C. Note that this does not prove that these breakthroughs caused AF maintenance, but rather that breakthroughs were observed in the more arrhythmogenic substrates.

The median AF cycle length was computed over the 12,669 sites both in the epicardium and in the endocardium. Over all simulations with more than 1 sec of AF, the cycle length was 144.8 ± 6.2 ms (epi) vs 144.9 ± 6.6 (endo) with the Workman model, and 258.4 ± 5.0 ms (epi) vs 258.6 ± 5.2 (endo) with the Kim model. The endoepicardial difference is physiologically negligible, although a paired t-test gave p<0.005 due to the large sample size.

Fig 2B–2G shows examples of membrane potential maps during AF. With moderate endoepicardial dissociation in the Workman model (panel B), endo- and epicardial depolarization waves were slightly shifted in space. Occasional breakthroughs were observed. More severe dissociation increased the distance between the endo- and epicardial wavefronts, providing space for more breakthroughs (panel C). Rarely, the epicardial wavefront near the core of a spiral was able to activate before the endocardial wavefront (panel D, yellow star). The Kim model led to similar observations (panel E). When the number of endoepicardial connections was strongly reduced (panel F), the colocalization of connected regions (outlined with black lines) and breakthroughs (e.g., yellow stars) became apparent. In these extreme cases, propagation in the epicardium was mostly the result of merging breakthroughs. At rare occasions (panel G), endocardial breakthroughs were identified when propagation was locally driven by an epicardial wave (yellow star).

**Fig 4:**
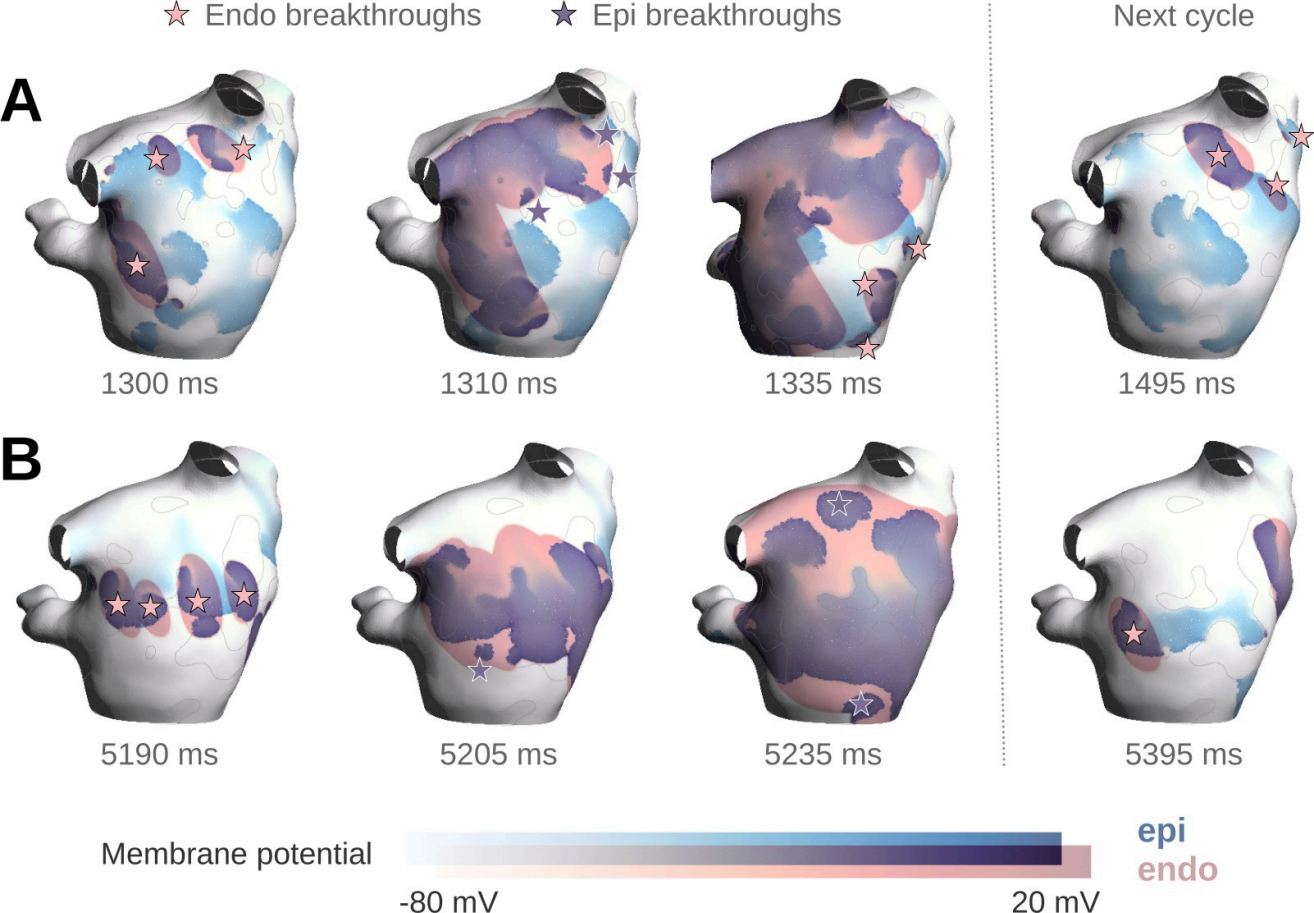
Two examples of transmural reentries. Membrane potential maps are color-coded as in Fig 2. Endocardial breakthroughs are indicated as a pink star and epicardial breakthroughs as a purple star. Parameter sets encoded as a quadruple (n_z_, #connected regions, σ_z_, fibrosis density) were as follows. Panel A: (2, 50, 0.3, 30%); Panel B: (2, 27, 0.4, 30%).

The occurrence of transmural reentrant circuits was sought. Since this type of reentry requires a consistent sequence of endocardial breakthroughs, we identified the simulations with at least 10 endocardial breakthroughs, each reaching the size of half the endocardial surface. There were 15 such simulations out of 4800, one with the Kim model and 14 with the Workman model (that has shorter wavelength), all with severe endoepicardial dissociation and fibrosis level. In these 15 cases, the average number of large endocardial breakthroughs was 22.1 ± 4.5 in the 5 s interval of analysis. Fig 4 shows two examples. The fibrillatory dynamics was driven by reentries in the epicardium; the activity in the endocardium was mainly composed of focal waves (pink stars), leading to a high level of dyssynchrony similar to the simulations of Gharaviri et al. [26]. These asynchronous focal waves eventually triggered epicardial breakthroughs (purple stars) and contributed to the complexity of the dynamics, although the resulting transmural circuits were not stable. The 15 cases were simulated for an additional 6 s with completely dissociated layers. In all these cases, fibrillation self-terminated in the endocardium, while it was sustained in the epicardium in 12 cases, suggesting that the reentrant circuits played here a limited role in AF maintenance.

### 3.3. Endoepicardial delays

**Fig 5:**
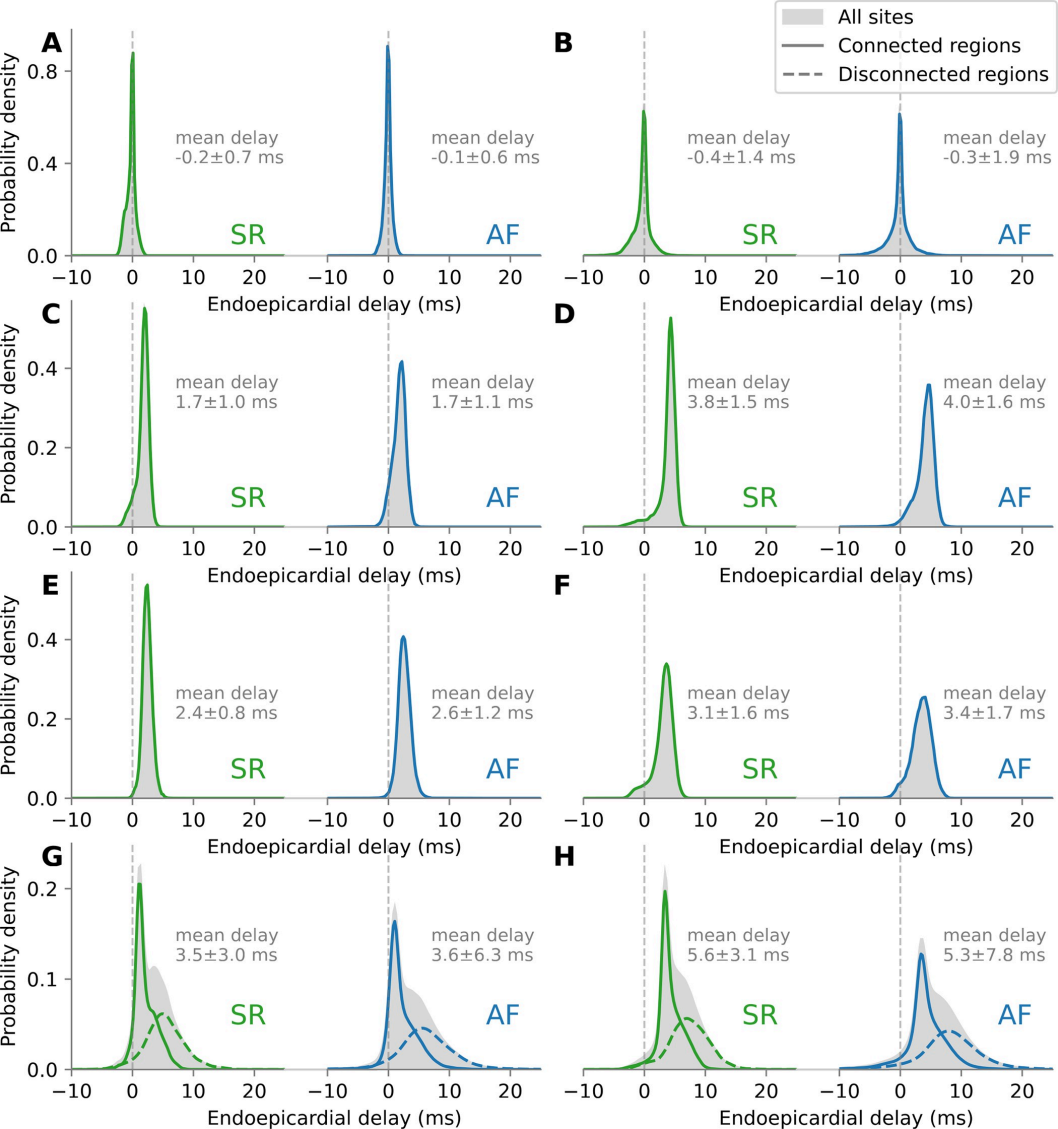
Examples of distributions of endoepicardial delays during sinus rhythm (SR) and atrial fibrillation (AF). Positive delay means that the endocardium is activated earlier. The distribution over all sites is shown in light gray. Mean and standard deviation of the delay is written next to each distribution. The Gaussian kernel-density estimate of the distribution over the sites in the connected (respectively disconnected) regions is displayed as solid (resp. dashed) curve. (A) uniform endoepicardial coupling (48k connected regions) without fibrosis, n_z_ = 2, σ_z_ = 0.3 mS/cm; (B) same as A, but with only 536 connected regions; (C) uniform coupling with 20% fibrosis, n_z_ = 4, σ_z_ = 0.4 mS/cm; (D) same as C, but with n_z_ = 8; (E) same as C, but with 30% fibrosis; (F) same C, but with σ_z_ = 0.2 mS/cm; (G) same as C, but with 191 connected regions; (H) same as G, but with n_z_ = 8.

For each simulation, endoepicardial delays were collected from all beats and all measurement sites. Fig 5 shows examples of such delay distributions during sinus rhythm and AF in the same substrate. Without fibrosis (Fig 5A and 5B), the distributions were relatively symmetric. Delays were caused by local interlayer differences in fiber orientation. Larger coupling heterogeneity (Fig 5B) increased the variance of the delay. Epicardial fibrosis (Fig 5C–5F) shifted the distributions toward positive delays (the endocardium activated earlier). Additional intermediate nodes (Fig 5D), more severe fibrosis (Fig 5E) and weaker transmural coupling (Fig 5F) resulted in longer delays. In the presence of heterogeneous layer dissociation (fewer endoepicardial connected regions and larger length scale), measurement sites in the connected and in the disconnected regions were separated to isolate their contribution to the delay distribution. This approach was inspired by the delay distribution analysis in Eckstein et al. [18]. In the disconnected regions (dashed curves), the delays were longer (up to 20 ms and beyond) as the epi- and endocardial wave fronts propagated independently at a different speed (Fig 5G and 5H). More severe fibrosis increased the conduction velocity ratio between the epi- and the endocardium, which allowed for even longer delays. Additional intermediate nodes had the same effect (Fig 5H). The delay distributions during sinus rhythm and AF were similar, only with slightly more variance during AF. The increase in variance during AF was more marked in the presence of heterogeneous layer dissociation (Fig 5G and 5H).

The mean and median of the delay were biased by the disconnected regions where the propagation of endo- and epicardial wave fronts were not causally related. In contrast, the mode of the delay distribution (the location of the peak) was a more robust measure that reflected the central tendency of the delays when the layers were functionally connected. Indeed, the difference between the mode of the full distribution (gray histograms in Fig 5) and that of the distribution based on the connected regions only (solid curves) was 0.11 ± 0.14 ms over all simulations. In the remaining subsections, we are going to focus on the substrates with fibrosis (≥ 10% density), because the effect of fibrosis on the delay dominated the effect of endoepicardial difference in fiber orientation, and therefore the mechanism causing that delay was more consistent across the substrates with fibrosis. Notably, the delay distributions were always unimodal, which was not always the case without fibrosis.

To recapitulate the factors that modulate the endoepicardial delay, an empirical regression formula for the mode of the delay was developed:

$$delay=\beta_{transmural}\frac{n_{z}}{\sqrt{\sigma_{z}/\sigma_{0}}(1+\gamma \sigma_{z}/\sigma_{0})}+\beta_{velocity}({CV}_{ratio}-1)+\frac{\beta_{dissoc}}{R_{regions}}+\beta_{intercept}$$

where σ_0_ = 1 mS/cm is a normalization factor, the four β are linear regression parameters and γ is a nonlinear regression parameter. The first term is intended to express the delay due to transmural propagation. Its numerator describes a distance and the square root formula in its denominator is proportional to the conduction velocity in the transmural cables (the γ term is a correction to conduction velocity in the low conductivity limit). The second term reflects the slower speed in the epicardium. Indeed, in the presence of endoepicardial dissociation, endo-epi difference in conduction velocity enables the accumulation of delays. The third term is associated with heterogeneous layer dissociation (the length scale of connected regions R_region_ is defined in section 2.3) and accounts for the fact that a breakthrough can reset the delay to zero. Finally, the last term is a bias.

**Fig 6:**
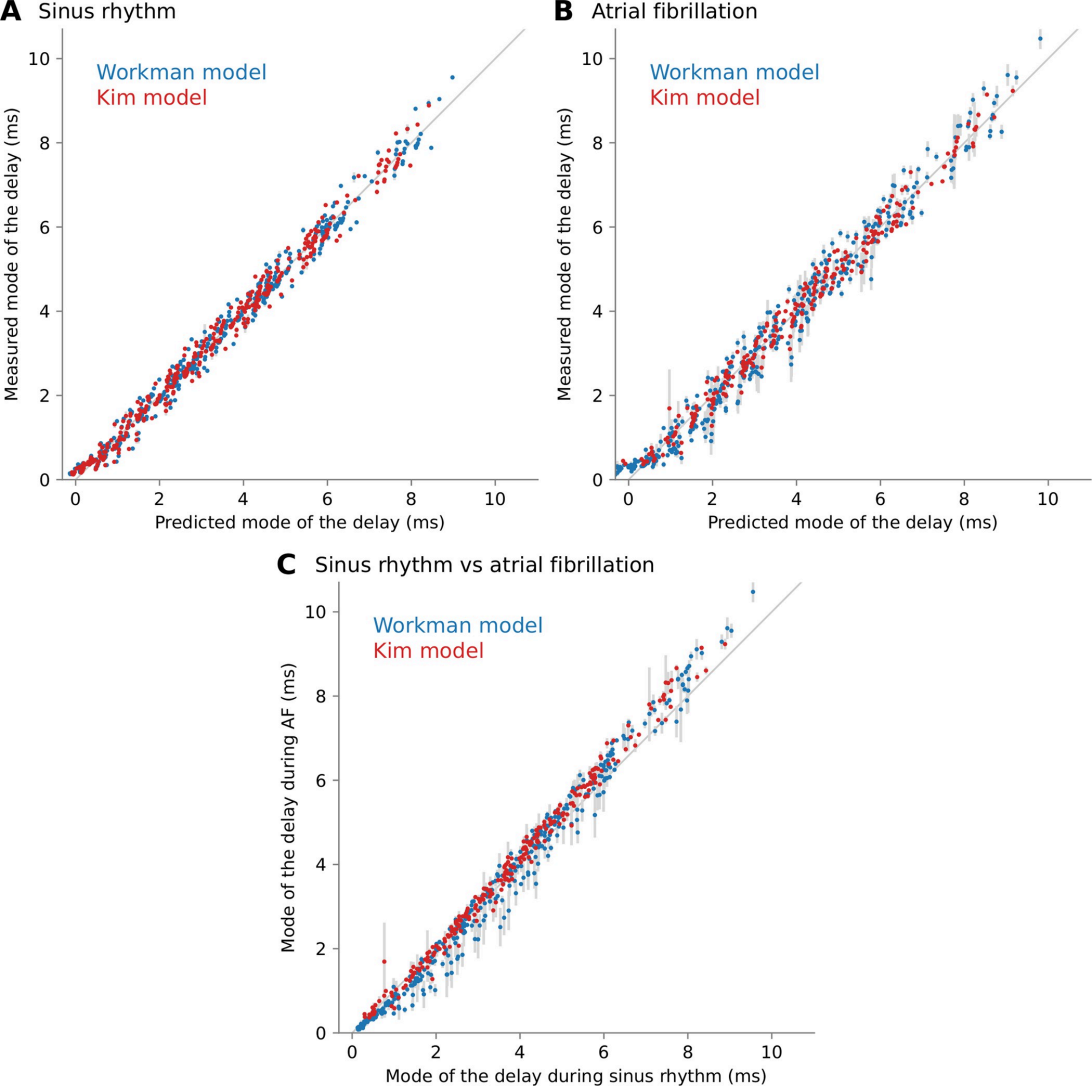
Measured and predicted mode of the endoepicardial delay distribution based on the regression formula. The membrane model is color coded. (A) Delays during sinus rhythm. Error bars indicate the standard deviation over 5 statistical realizations of the substrate. (B) Delays during atrial fibrillation. Error bars indicate the standard deviation over 5 fibrillatory initial conditions. (C) Measured model of the delay distribution in sinus rhythm vs atrial fibrillation.

The resulting estimated parameters are documented in Table 1. The predicted and measured delays are displayed in Fig 6A and 6B to illustrate the goodness-of-fit over the full observed range from 0 to 10 ms. The correlation coefficients were of the order of 0.99 with a root-mean square prediction errors in the range 0.2 to 0.4 ms (Table 1). As shown in Fig 6C, the modes of the delay in sinus rhythm and AF were similar (correlation coefficient: 0.994, root mean square difference: 0.32 ms). Despite large differences between the two cell models in terms of action potential duration restitution and cycle length, the regression parameters were similar. Nevertheless, note that these parameters were consistently larger with the Workman model. We postulate that this reflects the slightly slower conduction velocity of the Workman model, including in the transmural direction, which may promote longer delays.

**Table 1:** Regression parameters for the mode of the delay distribution.

	Workman		Kim	
	SR	AF	SR	AF
β_transmural_ (ms)	0.434 ± 0.003	0.475 ± 0.004	0.401 ± 0.002	0.440 ± 0.004
β_velocity_ (ms)	0.573 ± 0.035	1.045 ± 0.056	0.536 ± 0.034	0.878 ± 0.052
β_dissoc_ (ms mm)	0.233 ± 0.011	0.319 ± 0.017	0.211 ± 0.010	0.252 ± 0.014
β_intercept_ (ms)	-1.457 ± 0.038	-2.133 ± 0.059	-1.347 ± 0.034	-1.843 ± 0.063
γ	0.456 ± 0.003	0.569 ± 0.005	0.350 ± 0.003	0.411 ± 0.003
corr. coef.	0.994	0.988	0.995	0.992
RMSE (ms)	0.24	0.38	0.22	0.26

RMSE: root mean square error; corr. coef.: Pearson’s correlation coefficient; SR: sinus rhythm; AF: atrial fibrillation

The interpretation of the fitting formula is that the delay is increased by slow transmural propagation, large endoepicardial differences in conduction properties and more uniform interlayer coupling. The last effect is explained by the fact that the mode of the delay corresponds to connected regions where breakthrough can resynchronize the activation in both layers.

Another important descriptive statistic is the frequency of large delays. Endoepicardial asynchrony (EEA) was defined as the percentage of delays ≥ 15 ms as in de Groot et al. [17]. Table 2 documents EEA values separately for recordings in connected and disconnected regions for increasing degrees of dissociation. In the connected regions, EEA was low, indicating rare asynchrony when the layers were well connected. Layer dissociation enabled larger EEA to occur. The larger the disconnected region, the larger the EEA.

**Table 2:** Endoepicardial asynchrony (EEA; % of delays ≥ 15 ms) in the connected regions and in the disconnected regions, as well as all regions combined, for increasing levels of endoepicardial dissociation (number of connected regions, #conn.). EEA statistics were computed over all simulations in atrial fibrillation.

#conn.	EEA (connected regions)		EEA (disconnected regions)		EEA (combined)	
	Median (%)	Quartiles (%)	Median (%)	Quartiles (%)	Median (%)	Quartiles (%)
48k	0	0–0.1	0	0–0	0	0–0
6.5k	0.1	0–0.5	1	0–5	0	0–3
1.2k	1.2	0.1–4.6	7	1–31	2	0–20
536	1.8	0.4–6.8	15	5–49	6	1–22
301	2.2	0.6–7.6	26	7–59	8	2–31
191	2.3	0.8–6.7	35	15–67	8	2–40
50	2.1	0.9–4.1	65	46–79	17	2–65
27	1.2	0.7–2.8	72	53–80	18	1–72

### 3.4. Breakthrough activation patterns

**Fig 7:**
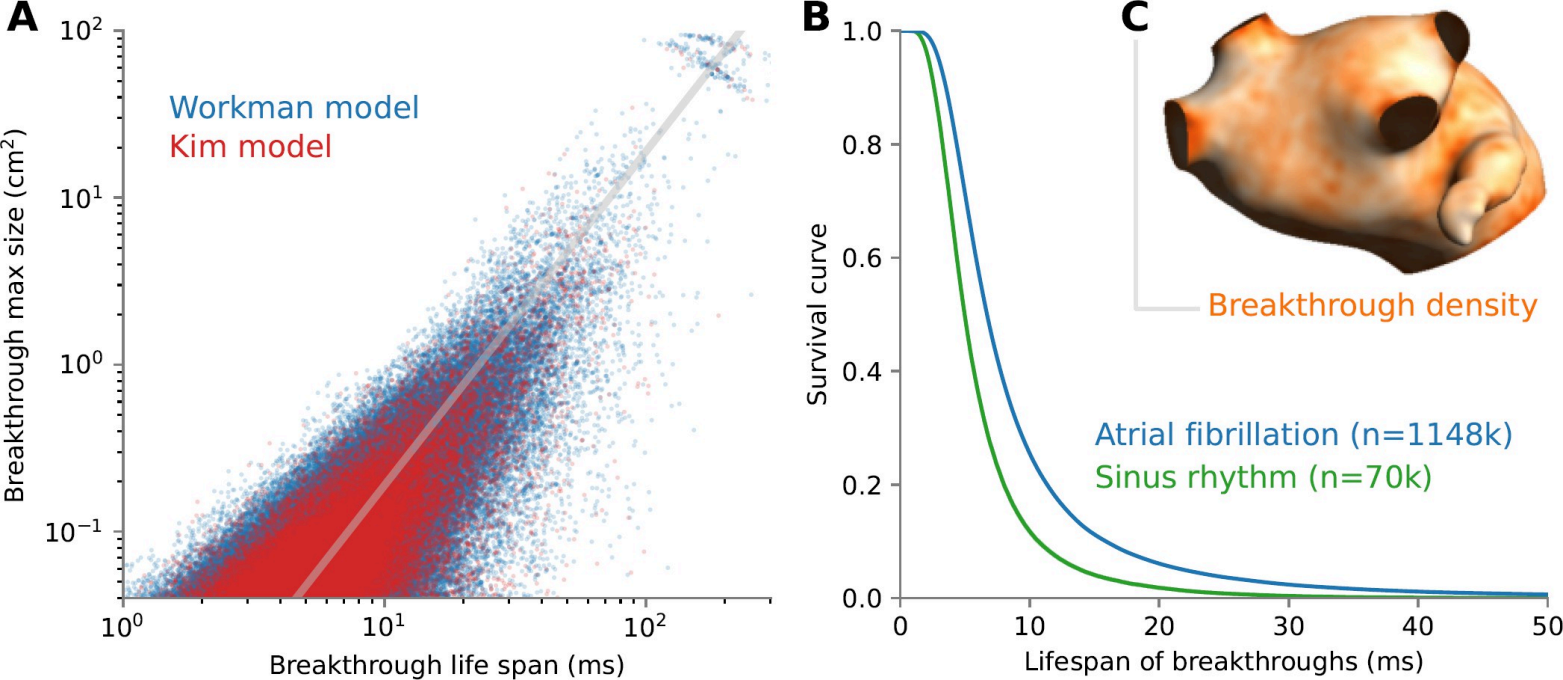
Lifespan and location of breakthroughs. (A) Scatter plot of life span vs maximum area for all breakthroughs in all simulations. Membrane model is color coded. The limits of the y-axis correspond to the minimum observable area (2 × 2 mm) and the total left atrium area. The diagonal gray line has a slope of 2 in logarithmic scale. (B) Survival curves of breakthroughs in sinus rhythm and atrial fibrillation, indicating their life span. The number of breakthroughs included is indicated in parentheses. (C) Map of breakthrough density averaged over all simulations during fibrillation. Density is color coded from 0 (white) to 1.5 breakthroughs per computational node (orange) after spatial filtering with a length scale of 2 mm.

A total of 1.2 million breakthroughs were identified according to our criteria over all simulations and the entire atrial surface; 95% of them were epicardial breakthroughs. Most of them were short-lived, lasting less than 10–20 ms. The maximum area that breakthrough waves covered before colliding with a spiral wave front was most often less than 1 cm^2^, although it sometimes reached more than half the atrial surface. Fig 7A shows that breakthrough wave maximum area is approximately proportional to the square of its lifespan, which is consistent with the propagation of a focal wave. The survival curve of breakthrough waves (Fig 7B) indicates that the median life span was 4.9 ms during sinus rhythm and 6.9 ms during AF. Breakthrough location was relatively uniformly distributed over the atrial surface. There tended to be, however, a higher density of breakthroughs near the border of the tissue such as veins and valve (Fig 7C), where fiber orientation was significantly different in each layer (Fig 1A).

**Fig 8:**
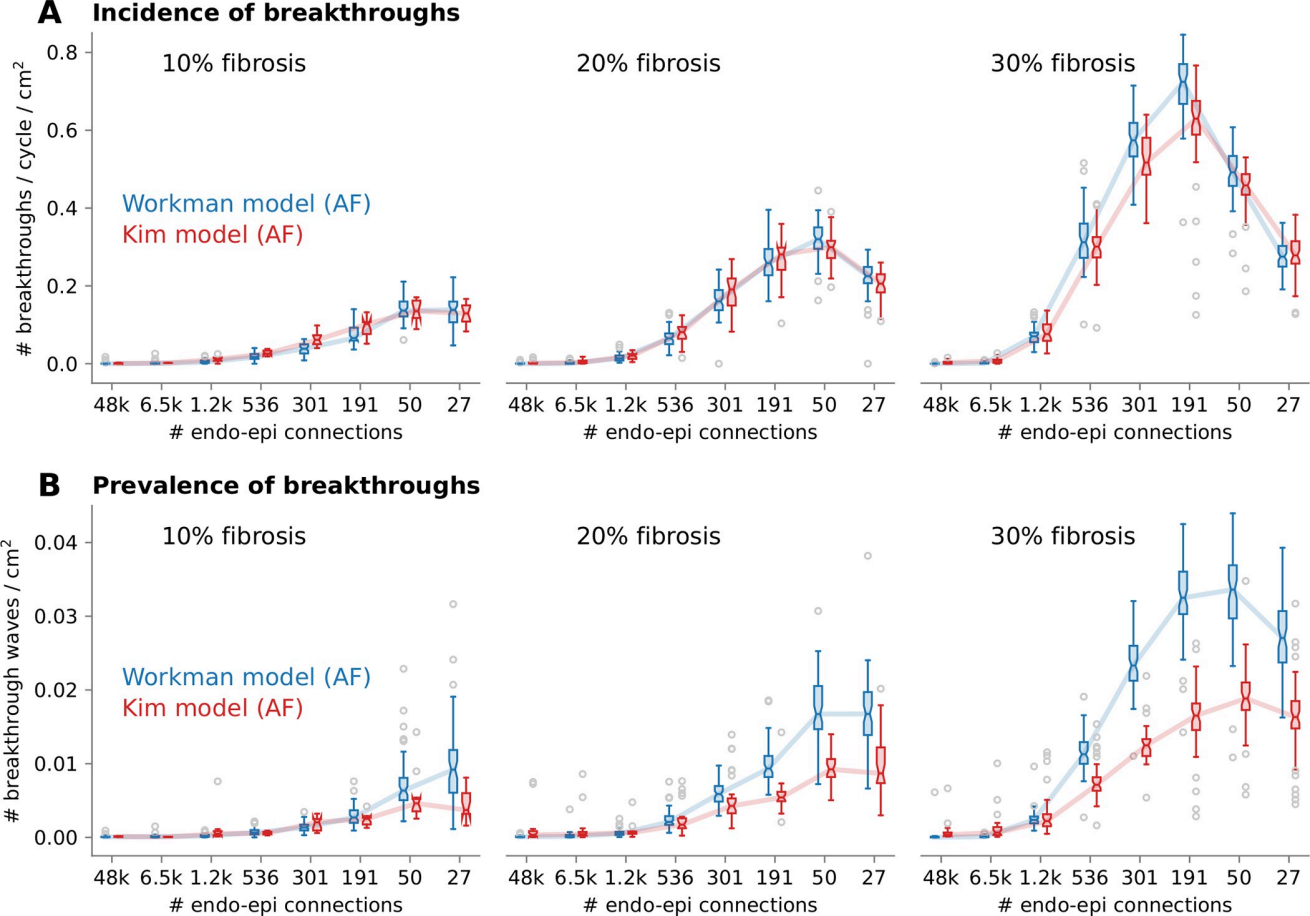
Incidence and prevalence of breakthroughs. Incidence (A) and prevalence (B) of breakthroughs during atrial fibrillation (AF) as a function of the level of endoepicardial dissociation heterogeneity (number of endoepicardial connections, as shown in Fig 1D), for epicardial fibrosis density from 10% (left) to 30% (right), and for both membrane models (color coded). Boxplots show the statistics over AF episodes with different values of n_z_ and σ_z_ of with different initial conditions. Small gray circles indicate outliers.

The incidence of breakthroughs depended on structural remodeling, more specifically layer dissociation as quantified by the number of endoepicardial connections, and much less on the fibrillatory dynamics or membrane model (Fig 8A). In a tissue with uniform interlayer coupling (> 1000 connections), very few breakthroughs were observed. As endoepicardial coupling became more heterogenous, the incidence of breakthroughs increased. In the extreme limit where the number of connections was very small (< 100), there were a limited number of potential breakthrough sites, which resulted in a decrease in the incidence of breakthroughs. Epicardial fibrosis promoted breakthroughs by allowing the buildup of larger delays in the disconnected regions.

The prevalence of breakthroughs (Fig 8B) also reflects breakthrough lifespan and AF cycle length. The prevalence of breakthroughs with the Workman model was larger because its cycle length was shorter. In the presence of severe degrees of heterogeneity, the order of magnitude of the prevalence was 0.01/cm^2^, which means that there was on average always one breakthrough wave somewhere on the atrial surface (101 cm^2^). The numerical values of incidence and prevalence of breakthroughs are affected by the threshold of 4 mm^2^ that we used for defining a potentially observable breakthrough (section 2.6). S1 Fig shows that breakthrough rates uniformly decrease when the threshold is doubled or tripled.

**Fig 9:**
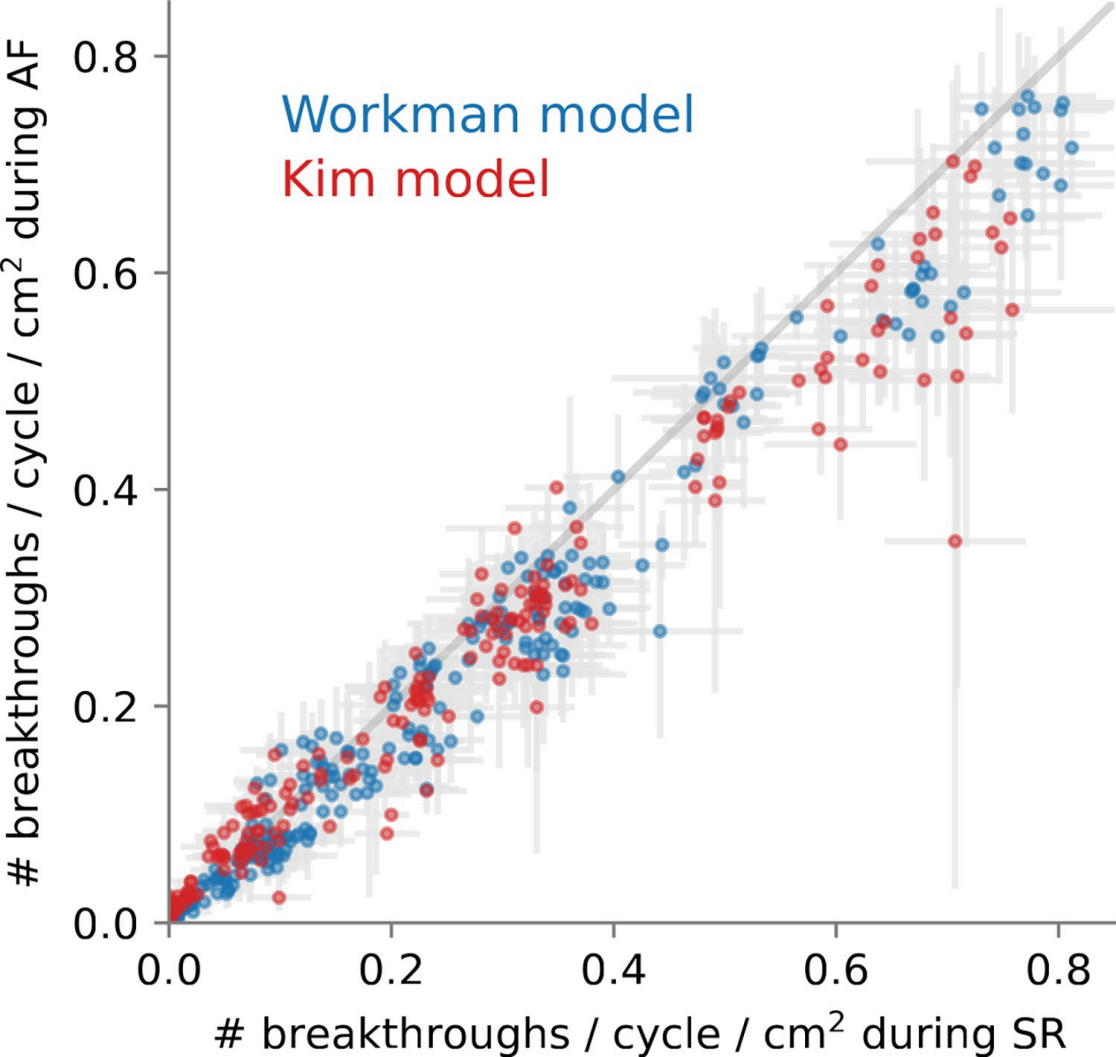
Correlation of the incidence of breakthrough during sinus rhythm (SR) and atrial fibrillation (AF) for both membrane model (color coded). Error bars indicate standard deviation over 5 statistical realizations of the substrate (SR) or 5 fibrillatory initial conditions (AF).

Because the incidence of breakthroughs is expressed per cycle, it can be compared during sinus rhythm and AF. Fig 9 shows that these incidences were correlated (corr. coef. 0.98). The incidence tended to be slightly larger during sinus rhythm (mean difference 0.02/cycle/cm^2^). This may be due to the tissue at rest in front of the depolarization wave where breakthroughs occurred. During AF, a partially refractory tissue is less susceptible to enable breakthroughs to propagate. Note that breakthroughs were still less frequent during sinus rhythm *in general* (0.14 ± 0.19 vs 0.17 ± 0.20 /cycle/cm^2^, t-test p < 0.01), because by construction, Fig 9 excludes the substrates (less likely to promote breakthroughs) in which AF initiation failed.

## 4. Discussion

### 4.1. Summary of our findings

We designed a bilayer model to study the consequences of endoepicardial dissociation on electrical propagation in the left atrium. Intermediate nodes were introduced between the two layers to extend the lower bound of interlayer coupling that can be simulated. In the presence of epicardial fibrosis, weak endoepicardial coupling (or, equivalently, a thicker atrial wall [30]) enabled propagation delays to appear. The delays were generally < 5 ms in the connected regions, reaching 10 ms for the most extreme values of the parameters. Longer delays were possible in the disconnected regions since propagation in the two layers was independent. The delays were longer with weaker coupling (low transmural conductivity and longer transmural pathway or larger n_z_) and higher fibrosis density. Propagation in sinus rhythm and AF in the same substrate were similar: depolarization waves were generally driven by the endocardium, and the delays did not accumulate over time. The very different AF dynamics and cycle lengths of the two cell models did not affect the conclusions.

Breakthroughs were observed in both sinus rhythm and AF, but they required severe heterogeneous endoepicardial dissociation and their lifespan was limited to 10–20 ms. In the absence of isolated endocardial bundles, breakthroughs occurred mostly when endoepicardial dissociation played that same role (disconnected region). Epicardial fibrosis only served as a mechanism to generate a difference in conduction velocity between the layers or pathways, like a fast-conducting fiber bundle would. As demonstrated by their short lifespan related to AF cycle length, breakthroughs locally perturbed propagation, but generally did not create three-dimensional reentries since the distance between two nearest transmural connections was generally shorter than half the wavelength, unlike in Gharaviri et al. [26]. The few counterexamples (Fig 4) were found in simulations with the most coarse-grained heterogeneous coupling distributions.

### 4.2. Comparison with clinical and experimental delays

Schuessler et al. [14] investigated the distribution of endoepicardial delays in dogs. Their published delay distributions looked like Fig 5A and 5B, with a mean delay generally < 1 ms and standard deviations between 3.5 and 9 ms. Over our 1200 simulations in sinus rhythm without fibrosis, the mode of the delay was -0.7 ± 1.9 ms and the standard deviation was 3.5 ± 1.6 ms, which is within the experimental range. In experiments, however, the difficulty to align endo- and epicardial electrodes and to determine activation time from electrograms may contribute to the variability in delays. The alignment issue can be mitigated by the use of a clamp-shaped high-density mapping device [15]. Accurately estimating short delays between electrograms, however, remains challenging [46, 47].

The criterion used in de Groot et al. [17] and van der Does et al. [48] for identifying asynchronous activation of the endo- and the epicardium was a transmural delay of more than 15 ms. Endo-epicardial asynchrony (EEA) was defined as the percentage of transmural delays larger than that 15 ms threshold. A group of patients had EEA < 12% and, in another group, EEA was in the range from 20 to 30%, with an outlier above 50%. In another study, Parameswaran et al. [16] found that 50.3% of the activation patterns in the right atrium showed significant EEA (transmural delay ≥ 20 ms in that paper) during persistent AF. In our simulations, 89% of AF episodes had an EEA < 12% in the connected regions, with the mode of the delay distribution ranging from 0 to 9 ms. On the other hand, in the disconnected regions, EEA covered the full range of percentages (Table 2), as illustrated by the delay distribution in Fig 5H. The two most severe degrees of dissociation (27 and 50 connections in Fig 1D) appear to produce EEA beyond typical clinical values and seem unlikely to occur *in vivo* except in localized areas.

Long endoepicardial delays indicating asynchronous propagation have been reported, notably in the right atrium. Van der Does et al. [48] measured delays in the range 4 to 84 ms (median: 13 ms) in a subgroup of patients. Parameswaran et al. [16] observed delays ≥ 40 ms in 28.6% of the activation patterns. Hansen et al. [24] found transmural activation delays in the right atrium up to 43 ± 22 ms, which enabled micro-anatomic intramural reentries, but in a thicker atrial wall with trabecular structures. Our model, in contrast, did not incorporate such three-dimensional structures, and, as a result, delays ≥ 40 ms were very rare, except for the two most severe degrees of dissociation.

### 4.3. Comparison with clinical and experimental breakthrough rates

**Fig 10:**
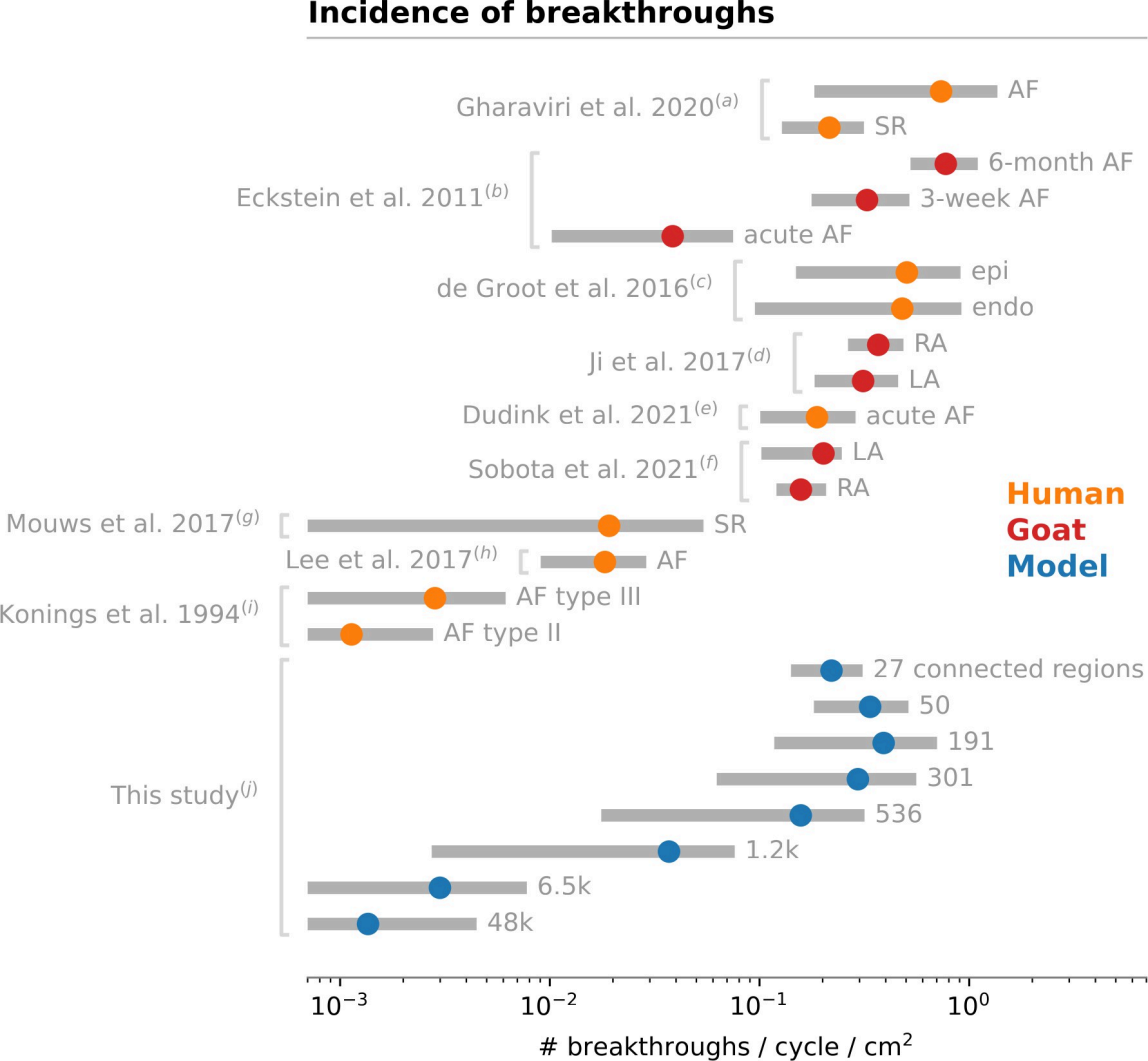
Ranges of breakthrough rates reported in clinical (orange) and experimental studies (red), compared to our simulations (blue). Mean ± standard deviation (unless stated otherwise) is shown in log scale. When needed, breakthroughs per cycle were calculated from breakthroughs per second using the mean cycle length. The mapping area used for normalization (if not indicated in the original paper) was estimated as the number of electrodes multiplied by the interelectrode distance squared. *Notes*: (a) recomputed from individual data [21]; (b) mapping area was calculated based on 90 electrodes [18]; (c) focal activations were excluded from breakthroughs [17]; (d) baseline data before drug injection [49]; (e) median ± half of interquartile range [50]; (f) median and quartiles [51]; (g) statistics computed from all histograms using a mean cycle length of 854 ms and 128 electrodes [22]; (h) based on repetitive nonrandom breakthroughs [52] and a mapping area of 92.85 cm^2^; (i) based on the percentages of breakthrough patterns [20]; (j) all data for each number of connected regions, Kim and Workman models combined. AF: atrial fibrillation; SR: sinus rhythm; RA: right atrium; LA: left atrium.

Calculating the breakthrough rate per cm^2^ of mapped area enables comparison across different species or computer models, experimental conditions, and mapping systems. Fig 10 shows the range of breakthrough rates calculated as best as possible from clinical and experimental data published in the literature, as well as breakthrough rates from our simulations.

Reported breakthrough rates span three orders of magnitude. There are multiple possible causes of variability in breakthrough rates. First, the progressive structural remodeling of the substrate tends to increase the breakthrough rate along time, as suggested by the study by Eckstein et al. [18] comparing acute AF, and 3 weeks and 6 months of AF in the goat. Earlier, Konings et al. [20] found a higher incidence of breakthrough patterns in type III (more complex) vs type I or II (simpler) human AF. On the other hand, breakthroughs have also been observed in sinus rhythm [21, 22]. Second, the location of the mapping area may matter. The trabecular structure in the right atrium certainly facilitates breakthroughs, as demonstrated by Hansen et al. [6, 24] using optical mapping in human tissue preparations. The pectinate muscles appear to play a role in the simulations of Gharaviri et al. [21]. Nevertheless, comparison of breakthrough rates between the right and the left atrium in goats has been non-conclusive [49, 51]. Further studies may prove otherwise. Third, breakthroughs may occur in the epi- or in the endocardium. Some studies only recorded signals in the epicardium; some did not distinguish between the two. In de Groot et al. [17], an equivalent number of epi- and endocardial breakthroughs were reported. Our simulations had mostly epicardial breakthroughs by design.

Technical factors can also affect the number of identified breakthroughs. Mapping density (interelectrode distance, from 1.5 to 5 mm in the cited references) has a direct impact on the minimal size of a detectable focal wave. Fig 7A gives a sense of how many breakthroughs would be hidden by a coarser mapping resolution. However, very small breakthroughs may not alter AF dynamics enough to be considered relevant. That was the rationale for our cutoff on wave size. The focal wave had to be sufficiently large (i.e. several passive space constants in diameter) to make it possible to validate that it was indeed propagating.

Another factor is that focal waves may be triggered by ectopic foci rather than by transmural propagation. This was never the case in our simulations, by design, but may happen in real tissue and is difficult to assess (particularly intermittent foci [52]) without simultaneous endo- and epicardial mapping. In de Groot et al. [17], about 66% of focal waves in the right atrial appendage were clearly identified as originating from transmural propagation (epi-to-endo or endo-to-epi). If this result can be extrapolated to other studies, this suggests that misclassification rate could be non-negligible when mapping only one side of the wall, but not sufficient to change the order of magnitude of the breakthrough rate.

In our simulations, breakthrough rates covered the full range of clinical and experimental data. The level of endoepicardial dissociation, quantified by the (inverse of the) number of connected regions, was a major factor (Fig 10), as well as fibrosis density (Fig 8).

### 4.4. Limitations

Our geometrical model was composed of two layers. Transmural propagation was possible only through mutually independent endoepicardial pathways (cables) whose length (n_z_) and conductivity (σ_z_) created an effect equivalent to wall thickness. The lack of lateral coupling between these transmural cables (Fig 1C) amplified endoepicardial delay (see in S2 Fig how impaired transmural conduction significantly aggravates the delay). This was an intentional choice to investigate the conditions needed to produce the large delays observed experimentally. Interestingly, the multilayer model by Chen et al. [25] introduced a highly fibrotic intermediate isolation layer, thus creating delayed propagation between discrete points of the epi- and the endocardium, which is reminiscent of our transmural cables.

In our previous study using the same geometry but with a cubic mesh (330 μm resolution) and wall thickness between 2 and 3 mm, delays up to 5 ms were simulated using a tissue conductivity up to 10 times smaller in the epicardium relative to the endocardium [30]. In contrast, our bilayer model generated simulations with delays of more than 9 ms. Part of the discrepancy comes from a different trade-off between spatial resolution within a layer versus the representation of interlayer coupling. The bilayer mesh constructed from cables aligned with fiber orientation or with the transmural axis enabled better control over transmural conduction properties. The model with n_z_ = 10 layers had only 76% more computational nodes than the pure bilayer model (n_z_ = 2), resulting in high efficiency as required for running thousands of large-scale simulations at 100 μm resolution. Extension to multiple layers as in Chen et al. [25] or to spatially varying values of n_z_ would be possible. However, spatial variations in thickness would introduce an additional independent variable when analyzing delay statistics. That was also the rationale for using a left atrium model, even if some of the experimental data we refer to have been collected in the right atrium.

Heterogeneity in endoepicardial dissociation was implemented as randomly distributed connected regions of different characteristic length scales. This approach generalizes the set of circular regions of fixed radius used previously [25, 26], with a control parameter describing the length scale of the spatial distribution. Information about pathophysiological distribution of endoepicardial dissociation in patients with persistent AF remains scarce. Also, fibrosis was present only in the epicardial layer to slow down propagation in one layer in a predictable way. To account for more symmetric outcome observed in several clinical studies [16, 17], the role of the epi- and endocardium could easily be exchanged in the model, and would lead to similar results. A more realistic model would include regions with predominantly epicardial and endocardial fibrosis. This would however weaken the analysis of the delay distributions since it would not be meaningful to pool the data from regions with different transmural fibrosis distribution.

Two membrane kinetics models were used, leading to different types of AF dynamics: a stable rotor with short cycle length and meandering waves with slower rates. Other AF mechanisms could affect both endoepicardial delays and the incidence of breakthroughs. Fiber bundles and interatrial connections provide additional pathways for micro- or macro-reentry and breakthroughs [21, 24, 38]. Patient-derived localized fibrosis patterns (instead of uniform diffuse fibrosis) could anchor the reentrant drivers [53, 54]. Focal AF maintained by ectopic foci generates focal activation patterns that might be confused with breakthroughs [17]. Neurogenic modulation by ganglionated plexi located on the epicardium might affect endoepicardial delays [55–57].

## 5. Conclusion

Our simulations establish the range of possible prevalence of endoepicardial delays and breakthroughs under the hypothesis of a weakly-coupled bilayer tissue with heterogeneous endoepicardial dissociation and an endocardial layer that was relatively uniform. Less extreme, or more physiologically realistic values of the model parameters might achieve similar prevalence if additional mechanisms were introduced, such as heterogeneous endocardial properties, regional differences in fibrosis or coupling, spatial variations in wall thickness and fiber bundles like in the right atrium or in the appendages. Considering the wide range of reported experimental and clinical values, our results may help determine what fraction of observed delays and breakthroughs might be explained by a thin bilayer with impaired transmural conduction, or if other mechanisms are likely to be involved.

## Supporting information

S1 Fig Effect of breakthrough minimum size on the incidence and the prevalence of breakthroughs. Fig 8 was redrawn using only breakthroughs whose maximum size (area) was larger than 8 mm^2^ (top panels) and 12 mm^2^ (bottom panels) instead of 4 mm^2^.
(PDF)

S2 Fig Impaired transmural propagation aggravates endoepicardial delays. To study in a simple model how adding intermediate layers (with propagation in the inner layers) would affect endoepicardial delays, we created four configurations of a ladder-shaped 2D model (0.1 mm resolution; 501 by 4 nodes; *right panels*). Endocardial conductivity was 4 mS/cm, transmural conductivity was 0.5 mS/cm, and epicardial was varied between 0.5 and 4 mS/cm. In the model with 4 layers, the top two layers were considered epicardium, and the bottom two layers were considered endocardium. Endoepicardial connections were spaced every 1, 2, 3 or 4 nodes. Left-to-right propagation was simulated and the endoepicardial delay (mean ± standard deviation) was calculated in the middle third of the tissue. *Left panel*: Endoepicardial delay as a function of the epicardial conductivity for different configurations. The fully coupled 4-layer model had the lowest delay. Removing propagation in the inner layers increased the delay. Extending the spacing between the endoepicardial connections resulted in a further prolongation of the delay.
(PDF)
