# Supplementary material for: Prevalence of endoepicardial asynchrony and breakthrough patterns in a bilayer computational model of heterogeneous endoepicardial dissociation in the left atrium
Source: PLoS One. 2024 Nov 22;19(11):e0314342. doi: 10.1371/journal.pone.0314342 (PMC11584087; doi:10.1371/journal.pone.0314342)
Supplement: S1 Fig — Fig 8 was redrawn using only breakthroughs whose maximum size (area) was larger than 8 mm2 (top panels) and 12 mm2 (bottom panels) instead of 4 mm2. (PDF) [file pone.0314342.s001.pdf]

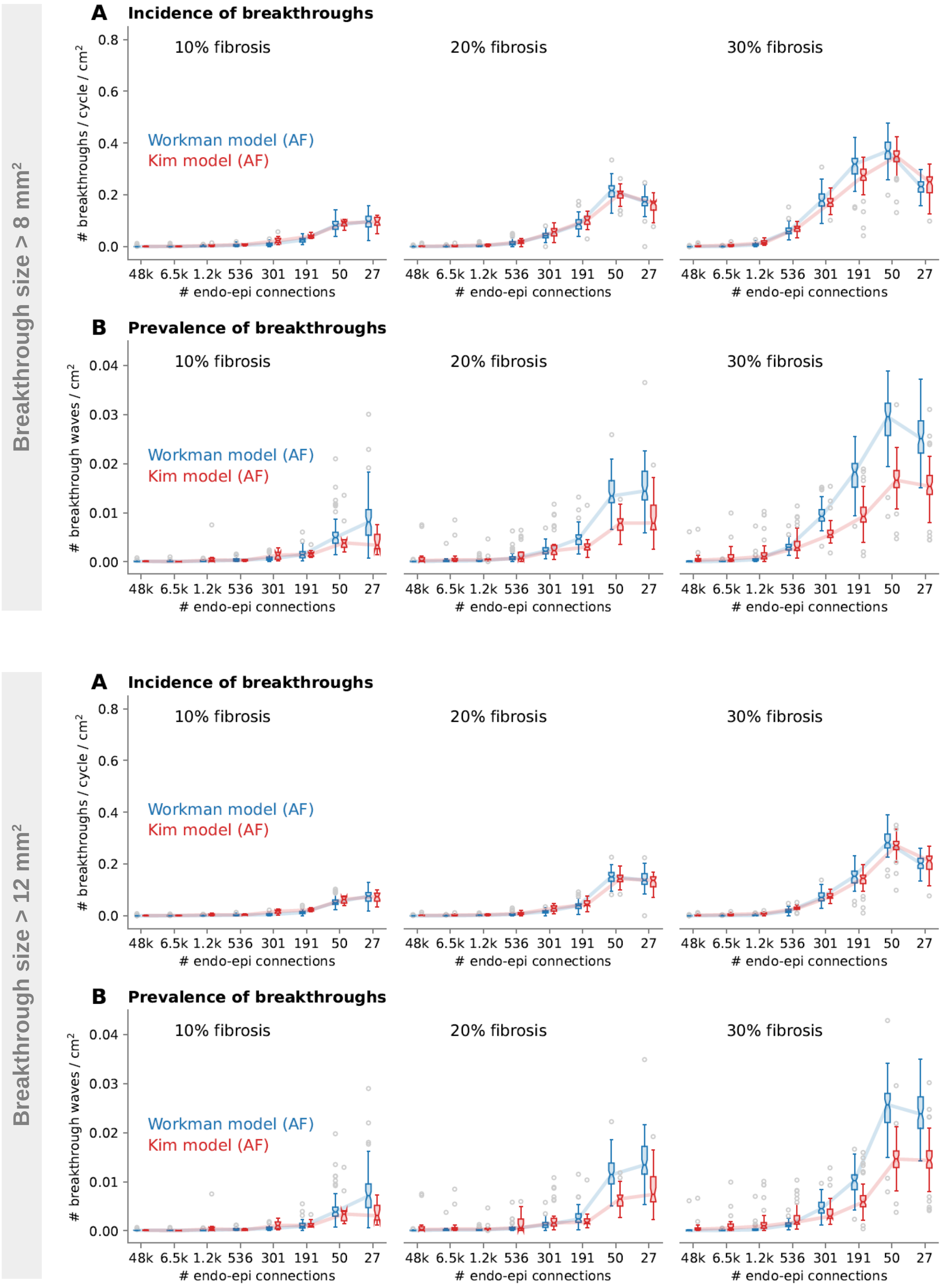
Breakthrough size > 8 mm²
A Incidence of breakthroughs
# breakthroughs / cycle / cm²
10% fibrosis
20% fibrosis
30% fibrosis
Workman model (AF)
Kim model (AF)
48k 6.5k 1.2k 536 301 191 50 27
# endo-epi connections
B Prevalence of breakthroughs
# breakthrough waves / cm²
10% fibrosis
20% fibrosis
30% fibrosis
Workman model (AF)
Kim model (AF)
48k 6.5k 1.2k 536 301 191 50 27
# endo-epi connections
Breakthrough size > 12 mm²
A Incidence of breakthroughs
# breakthroughs / cycle / cm²
10% fibrosis
20% fibrosis
30% fibrosis
Workman model (AF)
Kim model (AF)
48k 6.5k 1.2k 536 301 191 50 27
# endo-epi connections
B Prevalence of breakthroughs
# breakthrough waves / cm²
10% fibrosis
20% fibrosis
30% fibrosis
Workman model (AF)
Kim model (AF)
48k 6.5k 1.2k 536 301 191 50 27
# endo-epi connections
