# Supplementary figures and images for: Prevalence of endoepicardial asynchrony and breakthrough patterns in a bilayer computational model of heterogeneous endoepicardial dissociation in the left atrium
Source: PLoS One. 2024 Nov 22;19(11):e0314342. doi: 10.1371/journal.pone.0314342 (PMC11584087; doi:10.1371/journal.pone.0314342)

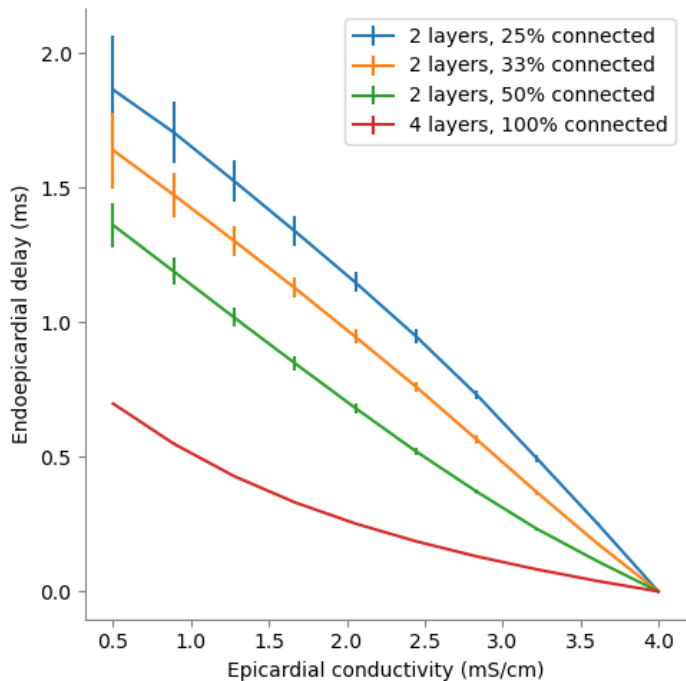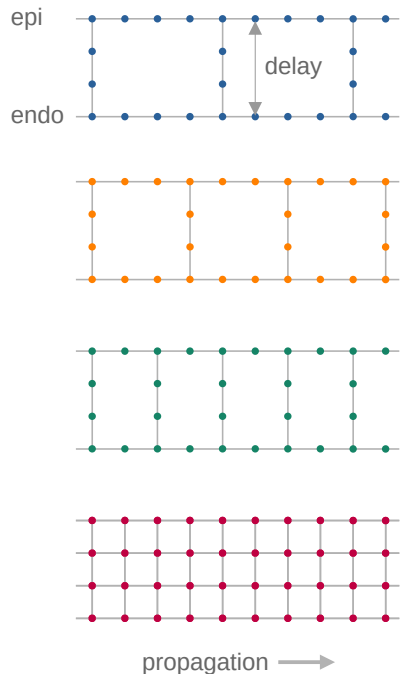

Supplement: S2 Fig — To study in a simple model how adding intermediate layers (with propagation in the inner layers) would affect endoepicardial delays, we created four configurations of a ladder-shaped 2D model (0.1 mm resolution; 501 by 4 nodes; right panels). Endocardial conductivity was 4 mS/cm, transmural conductivity was 0.5 mS/cm, and epicardial was varied between 0.5 and 4 mS/cm. In the model with 4 layers, the top two layers were considered epicardium, and the bottom two layers were considered endocardium. Endoepicardial connections were spaced every 1, 2, 3 or 4 nodes. Left-to-right propagation was simulated and the endoepicardial delay (mean ± standard deviation) was calculated in the middle third of the tissue. Left panel: Endoepicardial delay as a function of the epicardial conductivity for different configurations. The fully coupled 4-layer model had the lowest delay. Removing propagation in the inner layers increased the delay. Extending the spacing between the endoepicardial connections resulted in a further prolongation of the delay. (PDF) [file pone.0314342.s002.pdf]
